# Loss of ERα partially reverses the effects of maternal high-fat diet on energy homeostasis in female mice

**DOI:** 10.1038/s41598-017-06560-x

**Published:** 2017-07-25

**Authors:** Troy A. Roepke, Ali Yasrebi, Alejandra Villalobos, Elizabeth A. Krumm, Jennifer A. Yang, Kyle J. Mamounis

**Affiliations:** 1 Department of Animal Sciences, School of Environmental and Biological Sciences, Rutgers, The State University of New Jersey, New Brunswick, NJ USA; 2 Graduate Program in Endocrinology and Animal Biosciences, Rutgers, The State University of New Jersey, New Brunswick, NJ USA; 3 Nutritional Sciences Graduate Program, Rutgers, The State University of New Jersey, New Brunswick, NJ USA; 4 New Jersey Institute for Food, Nutrition, and Health, Rutgers, The State University of New Jersey, New Brunswick, NJ USA; 5 Present Address: Department of Reproductive Medicine, University of California, San Diego, San Diego CA 92103 USA; 6 Present Address: Burnett School of Biomedical Sciences, College of Medicine, University of Central Florida, Orlando, FL 32827 USA

## Abstract

Maternal high-fat diet (HFD) alters hypothalamic developmental programming and disrupts offspring energy homeostasis in rodents. 17β-estradiol (E2) also influences hypothalamic programming through estrogen receptor (ER) α. Therefore, we hypothesized that females lacking ERα would be more susceptible to maternal HFD. To address this question, heterozygous ERα knockout (WT/KO) dams were fed a control breeder chow diet (25% fat) or a semi-purified HFD (45% fat) 4 weeks prior to mating with WT/KO males or heterozygous males with an ERα DNA-binding domain mutation knocked in (WT/KI) to produce WT, ERα KO, or ERα KIKO females lacking ERE-dependent ERα signaling. Maternal HFD increased body weight in WT and KIKO, in part, due to increased adiposity and daytime carbohydrate utilization in WT and KIKO, while increasing nighttime fat utilization in KO. Maternal HFD also increased plasma leptin, IL-6, and MCP-1 in WT and increased arcuate expression of *Kiss1* and *Esr1* (ERα) and liver expression of *G6pc* and *Pepck* in WT and KIKO. Contrary to our hypothesis, these data suggest that loss of ERα signaling blocks the influence of maternal HFD on energy homeostasis, inflammation, and hypothalamic and liver gene expression and that restoration of ERE-independent ERα signaling partially reestablishes susceptibility to maternal HFD.

## Introduction

Because maternal influences can impact physiological trait expression, the consequences of the obesity epidemic in reproductive-age women are borne by the next generation through alterations in maternal programming of fetal and neonatal development. Indeed, it is estimated that ~35–40% of reproductive-age women are obese or overweight in the USA^1^. The current idea of intergenerational links between maternal nutrition and health began with the “Thrifty Gene” hypothesis proposed by David J.P. Barker^2^. Stated simply, a poor nutritional environment during pregnancy, lactation, and early infancy predisposes offspring whose adult nutritional environment is richer than the gestational diet to chronic diseases including ischemic heart disease, stroke, hypertension, Type II DM, and obesity^2^.

Recent studies on maternal obesity or the effect of maternal high-fat diet (HFD) have demonstrated similar effects on offspring energy homeostasis. These studies found lower birth weights in treated offspring compared to control offspring followed by a catch-up weight gain, adult obesity, and insulin resistance, especially on an obesogenic diet^3–5^. Other studies showed higher birth and adult weights in offspring of diet-induced obesity (DIO) dams compared to control offspring^6^. Although the molecular mechanisms underlying the effects of maternal HFD are still being explored, changes in hypothalamic gene expression, melanocortin circuitry, neurogenesis, and neuroinflammation have emerged as central mediators of pathogenesis^7–11^. For example, maternal HFD stimulates hypothalamic neurogenesis of orexigenic neuropeptide Y (NPY) neurons and suppresses anorexigenic proopiomelanocortin (POMC) neurons in male offspring^12^, which favors hyperphagia. Maternal HFD also hypermethylates the POMC promoter in the hypothalamus of female offspring, which potentially reduces expression of the gene, leading to an increase in food intake and a reduction in energy expenditure^13^.

The reproductive steroid 17β-estradiol (E2) regulates various aspects of energy homeostasis through both peripheral actions and central mechanisms. The key brain regions that mediate the effects of E2 on energy homeostasis are the hypothalamus and the hindbrain^14–17^ wherein E2 suppresses feeding and augments energy expenditure and activity primarily through estrogen receptor (ER) α^18, 19^. Indeed, ERα knockouts (KO) exhibit an obese phenotype with increased visceral adiposity and decreased energy expenditure^20, 21^.

ERα signaling functions through nuclear-initiated and membrane-initiated signaling. To control gene expression, nuclear-initiated ERα signaling binds to DNA directly through the estrogen response elements (ERE) or through ERE-independent mechanisms, such as protein-protein interactions with other transcription factors^22^. ERα can also activate membrane-initiated signaling cascades (MAPK, PLC, PI3K) to modulate cell physiology and control gene expression^23–28^. The restoration of ERE-independent signaling (both membrane- and nuclear-initiated) in ERα KO female mice normalizes energy homeostasis. These females, called ERα KIKO, express an ERα that does not bind to ERE but retains nuclear-initiated tethered transcriptional regulation and membrane-initiated activation of signaling cascades. Adult KIKO females do not become obese or glucose intolerant, suggesting that ERE-independent ERα signaling is sufficient for the normal development and maintenance of energy and glucose homeostasis^29, 30^. Thus, a potential basis for the disruption in energy homeostasis in KO females is the loss of ERE-independent ERα signaling during neurogenesis^31–33^ and the proliferation and differentiation of neural stem cells^34^.

Because the loss of ERα and the influence of maternal HFD alters hypothalamic developmental programming leading to dysregulation of energy homeostasis, we hypothesized that the total loss of ERα would make female mice more susceptible to the effects of maternal HFD. Furthermore, because ERE-independent ERα signaling restores normal energy homeostasis, we also hypothesized that ERE-independent ERα signaling would be protective against the effects of maternal HFD. To address these hypotheses, we employed a standard maternal HFD paradigm using heterozygous dams mated to heterozygous males and followed their WT, KO, and KIKO female offspring into adulthood.

## Results

### Body weight and body composition

**Figure 1:**
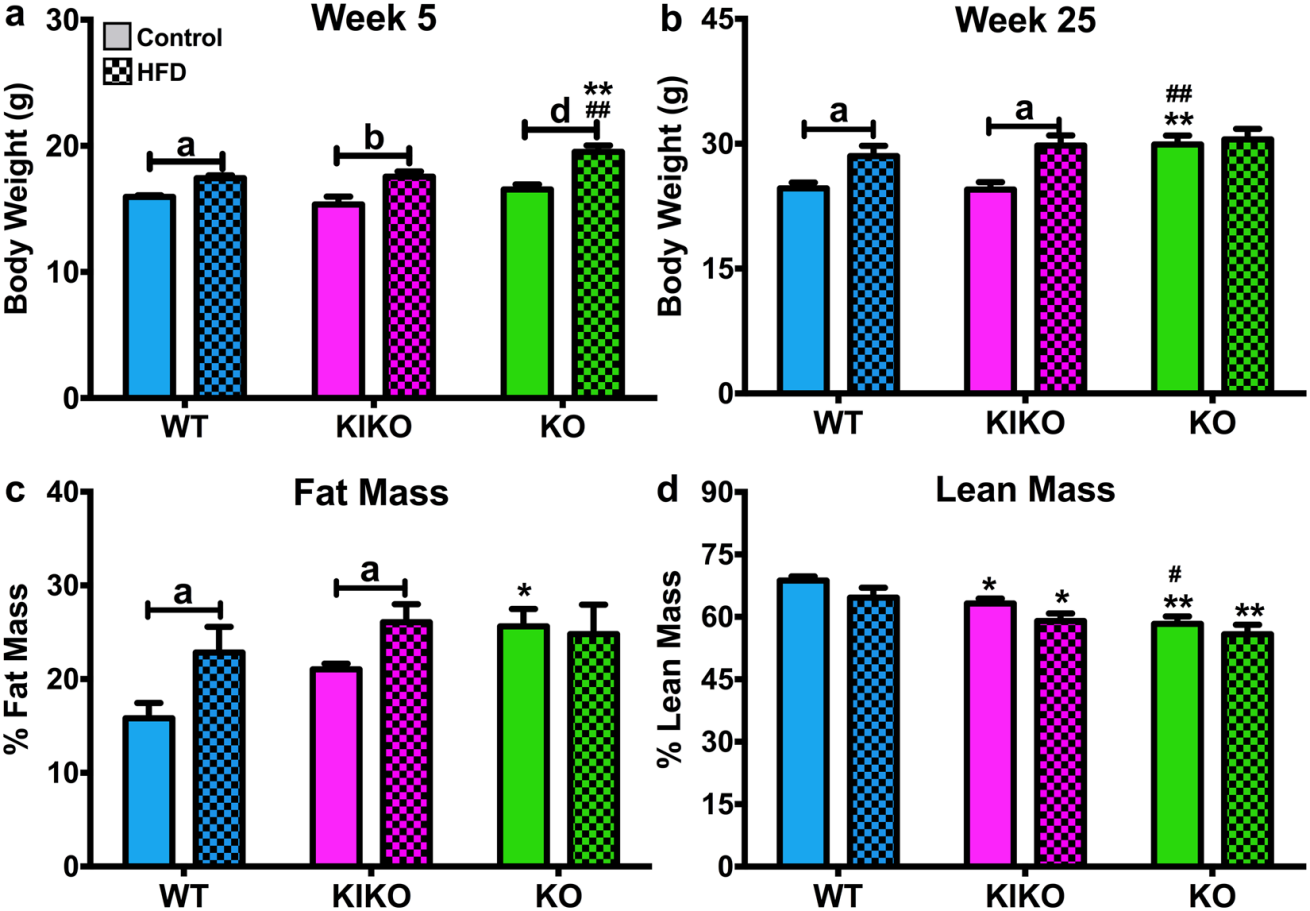
Body weight and body composition of adult females. (**a**) Body weights at week 5 in all genotypes from Control-fed and HFD-fed dams. (**b**) Body weights at week 25 in all genotypes after 20 weeks of a low-fat chow diet. (**c**) Percent body fat (fat mass/body mass) of female mice from all groups. (**d**) Percent lean mass (lean mass/body mass) of female mice from all groups. Control = maternal control diet and HFD = maternal HFD. Data were analyzed by two-way ANOVA with *post-hoc* Newman-Keuls test. Sample sizes were 9 to 12 per genotype per treatment and data are expressed as mean ± SEM. Capped lines denote comparison between maternal diets within genotypes. Asterisks (*) denote comparison to WT within the same diet group. The pound sign (**#**) denotes comparison of KIKO and KO within the diet group. (a/*/# = P < 0.05; b/**/## = P < 0.01; c/***/### = P < 0.001; d/****/#### = P < 0.0001).

By week 5 (peripubertal), females from HFD-fed dams of each genotype weighed more than their counterparts from control (Con)-fed dams (Fig. 1a). WT from Con-fed dams (*n* = 11) weighed 15.9 ± 0.12 g, and WT from HFD-fed dams (*n* = 11) weighed 17.4 ± 0.2 g (P < 0.05). KIKO from Con-fed dams (*n* = 9) weighed 15.3 ± 0.6 g, and KIKO from HFD-fed dams (*n* = 9) weighed 17.6 ± 0.4 g (P < 0.01). KO from Con-fed dams (*n* = 9) weighed 16.5 ± 0.4 g, and KO from HFD-fed dams (*n* = 12) weighed 19.5 ± 0.5 g (P < 0.001). However, this effect of maternal HFD was lost in KO females by week 9 (post-puberty) while WT and KIKO females from HFD-fed dams were slightly heavier than WT and KIKO from Con-fed dams throughout the study (data not shown).

After 23 weeks on a standard (low-fat) chow diet (Fig. 1b), WT from Con-fed dams (*n* = 11) weighed 24.6 ± 0.7 g, and WT from HFD-fed dams (*n* = 11) weighed 28.5 ± 1.2 g (P < 0.05). KIKO from Con-fed dams (*n* = 9) weighed 24.5 ± 0.9 g, and KIKO from HFD-fed dams (*n* = 9) weighed 29.0 ± 1.5 g (P < 0.05). KO from Con-fed dams (*n* = 9) weighed 29.9 ± 1.6 g, and KO from HFD-fed dams (*n* = 12) weighed 30.5 ± 1.3 g (*ns*). In summary, KO from Con-fed dams weighed more than their WT and KIKO counterparts. However, maternal HFD increased body weight in WT and KIKO and not in KO females. Collectively, these data suggest that the loss of ERE-dependent signaling in KO abrogates the effects of maternal HFD.

Body fat accumulation (% fat mass) in the Con-fed females was similar to our previous study (32) with KO fatter than WT (P < 0.5) but not KIKO. Maternal HFD increased body fat in WT (P < 0.01) and KIKO (P < 0.05; Fig. 1c), indicating that increased deposition of adipose tissue underlies the increase in body weight for WT and KIKO from HFD-fed dams. KO from Con-fed females had less lean mass than WT (P < 0.01) and KIKO (P < 0.05), which also had less lean mass than WT (P < 0.05; Fig. 1d). WT from HFD-fed dams had more lean mass than both KIKO (P < 0.05) and KO (P < 0.01).

Food intake was measured for the Con-fed females in all genotypes for 1 week in single-housing cages. As previously reported^32^, WT consumed more food than KIKO or KO during the weeklong trial. Average food intake for the week was 22.7 ± 1.8 g in WT, 18.3 ± 0.7 g in KIKO (P < 0.05), and 18.1 ± 1.3 g in KO (P < 0.05), which corroborates our previous findings^35^ (data not shown). However, we observed a loss of body weight in KIKO and KO during the week, most likely due to the stress of single housing. Therefore, all control females were placed back in group-housed cages and allowed to recover body weights prior to glucose and insulin tolerance testing. We did not examine food intake in females from HFD-fed dams due to concerns that the short-term feeding studies would be confounded by the stress of single housing.

### Metabolic parameters

**Figure 2:**
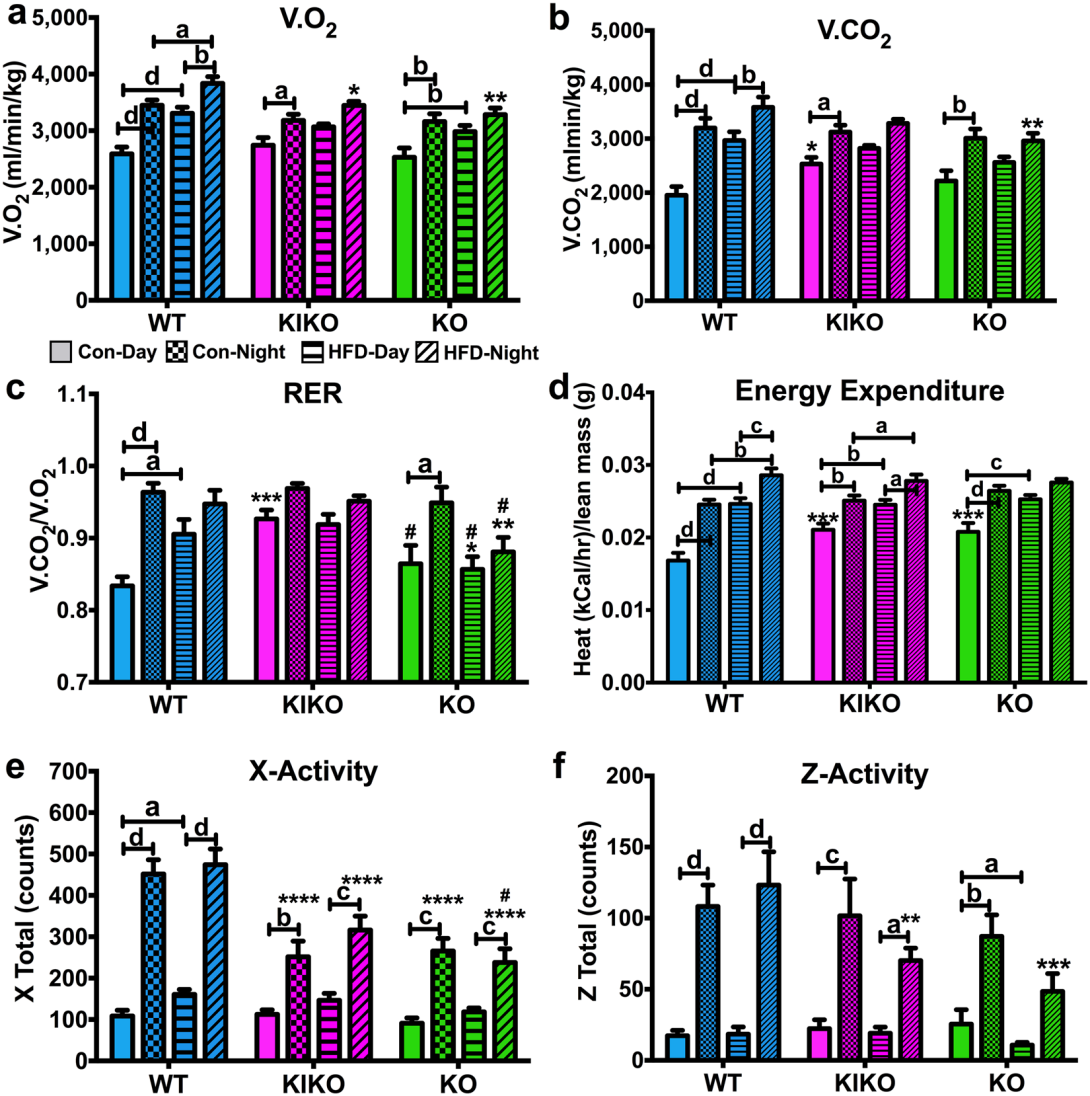
Metabolic and activity parameters in females from all genotypes after 20 weeks of adult chow diet determined using the CLAMS. (**a**) V.O_2_ (ml/min/kg); (**b**) V.CO_2_ (ml/min/kg); (**c**) Respiratory exchange ratio (RER) (V.CO_2_ /V.O_2_); (**d**) Energy expenditure (kCal/hr/lean mass (g)); (**e**) X-plane activity (counts); and (**f**) Z-plane activity (counts). Data were analyzed by a multi-factorial ANOVA (genotype, maternal diet, time) with *post-hoc* Newman-Keuls test. See Fig. 1 for information on treatment categories, sample sizes, and statistical comparisons (a/*/# = P < 0.05; b/**/## = P < 0.01; c/***/### = P < 0.001; d/****/#### = P < 0.0001).

To determine the effects of maternal HFD on energy expenditure, substrate utilization, and activity, all females were transferred to a Comprehensive Lab Animal Monitoring System (CLAMS) unit for 48 h using data only from the last 24 h to calculate metabolic parameters and activity^36^. V.O_2_ was affected by genotype and maternal HFD (Fig. 2a). During the day, V.O_2_ was elevated by maternal HFD in WT (P < 0.0001) and KO (P < 0.01), and during the night, V.O_2_ was elevated by maternal HFD only in WT (P < 0.05). WT females from HFD-fed dams exhibited higher nighttime V.O_2_ than their KIKO (P < 0.05) and KO (P < 0.01) counterparts. Maternal HFD eliminated the differences between nighttime and daytime V.O_2_ in KIKO and KO females.

V.CO_2_ was affected by genotype, maternal HFD, and time (Fig. 2b). Maternal HFD elevated daytime V.CO_2_ in WT (P < 0.0001) but not in KIKO or KO. V.CO_2_ was elevated at nighttime compared to daytime in all genotypes (WT: P < 0.0001; KIKO: P < 0.05; KO: P < 0.01) from Con-fed dams. Similar to V.O_2_, maternal HFD eliminated this elevation during the night in KIKO and KO. Daytime V.CO_2_ in KIKO from Con-fed dams was elevated compared to WT (P < 0.05), and nighttime V.CO_2_ in KO from HFD-fed dams was lower than WT (P < 0.01).

Respiratory exchange ratio (RER) was affected by genotype, time, and the interaction of genotype and maternal HFD (Fig. 2c). Daytime RER was elevated by maternal HFD in WT (P < 0.05) but not in KIKO or KO. As we have previously reported, daytime RER in KIKO from Con-fed dams was higher than in WT (P < 0.001) or KO (P < 0.05), indicating that KIKO females preferentially utilize carbohydrates during the day compared to both WT and KO. Hence, nighttime RER was not higher in KIKO from Con-fed dams as was found in WT and KO. Interestingly, RER in KO was generally lower than both WT and KIKO except for nighttime RER in KO from Con-fed dams. Because body weight can influence metabolism, RER was analyzed by an analysis of covariance (ANCOVA) with body weight as a covariate and plotted as a function of body weight to illustrate these effects (Supplemental Figure S2a). Overall, neither genotype nor maternal HFD affected the relationship of body weight and RER.

Heat production (energy expenditure) normalized to lean body mass was affected by genotype, maternal diet, time, and interactions of genotype and maternal diet and maternal diet and time (Fig. 2d). In both maternal diets, heat was elevated in the nighttime compared to the daytime in WT and KIKO, but only KO from Con-fed dams. Unlike V.O_2_, V.CO_2_, and RER, maternal HFD elevated heat production in WT and KIKO during both time periods, but only during the daytime in KO. Finally, daytime heat production in KIKO and KO from Con-fed dams was higher compared to WT (P < 0.05 for both). Elevation of heat production indicates higher metabolic rates, thus maternal HFD augmented metabolic rates only during the daytime and independent of activity in WT and KIKO. We also analyzed daytime and nighttime heat by an analysis of covariance (ANCOVA) with body weight as a covariate (Supplemental Figure S2b and c). As expected, maternal HFD affected the relationship of body weight and heat during the day (P < 0.0001) and night (P < 0.0001), although there was an interaction between genotype and maternal diet (P < 0.05) during the daytime.

Both X-plane and Z-plane activity were affected by genotype, time, and the interactions between genotype and time, but only X activity was affected by maternal HFD (Fig. 2e and f). X-plane activity was higher in the nighttime than the daytime in all genotypes, regardless of maternal diet. However, both KIKO and KO females were less active in the nighttime compared to WT, regardless of maternal diet, as previously reported^35^. Interestingly, there was a subtle but significant increase in daytime activity in WT due to maternal HFD (P < 0.05). Z-plane activity was higher in the nighttime than the daytime for all genotypes, regardless of diet. However, maternal HFD reduced daytime Z-plane activity in KO and reduced nighttime Z-plane activity in KIKO (P < 0.01) and KO (P < 0.001) compared to WT. These data suggest that ERE-dependent ERα signaling is necessary for the maintenance of normal activity in female mice.

### Glucose and insulin tolerance

**Figure 3:**
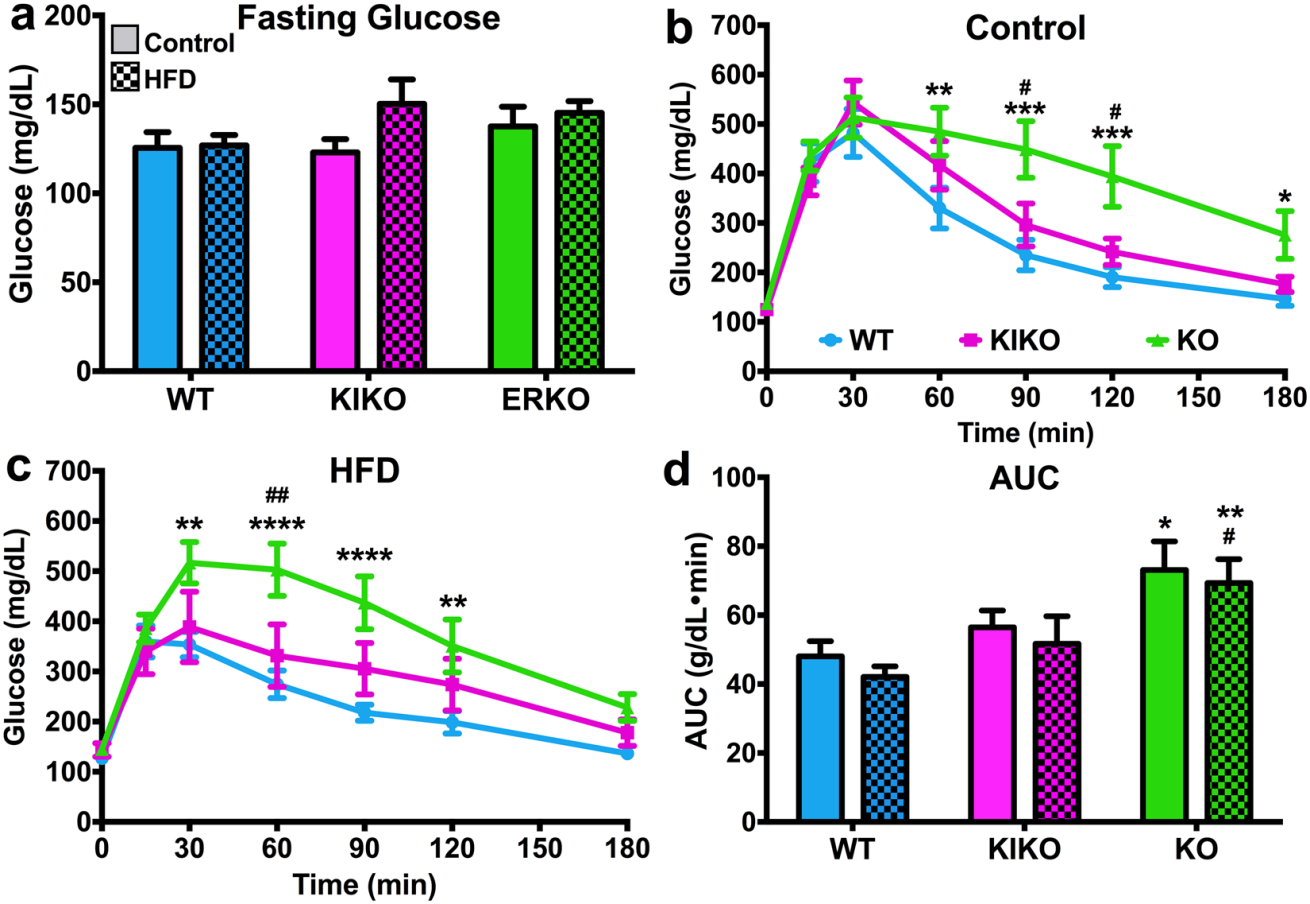
Fasting glucose levels and glucose tolerance test (GTT) in adult females from all genotypes after 20 weeks of adult chow diet. (**a**) Fasting glucose levels. Results from the GTT from (**b**) all genotypes from Control-fed dams and (**c**) all genotypes from HFD-fed dams. (**d**) Area under the curve (AUC) analysis for all genotypes from both maternal diets. (**a** and **d**) Data were analyzed by a two-way ANOVA with *post-hoc* Newman-Keuls test. (**b** and **c**) Data were analyzed by repeated-measures, multi-factorial ANOVA with *post-hoc* Newman-Keuls test. See Fig. 1 for information on treatment categories, sample sizes, and statistical comparisons (a/*/# = P < 0.05; b/**/## = P < 0.01; c/***/### = P < 0.001; d/****/#### = P < 0.0001).

**Figure 4:**
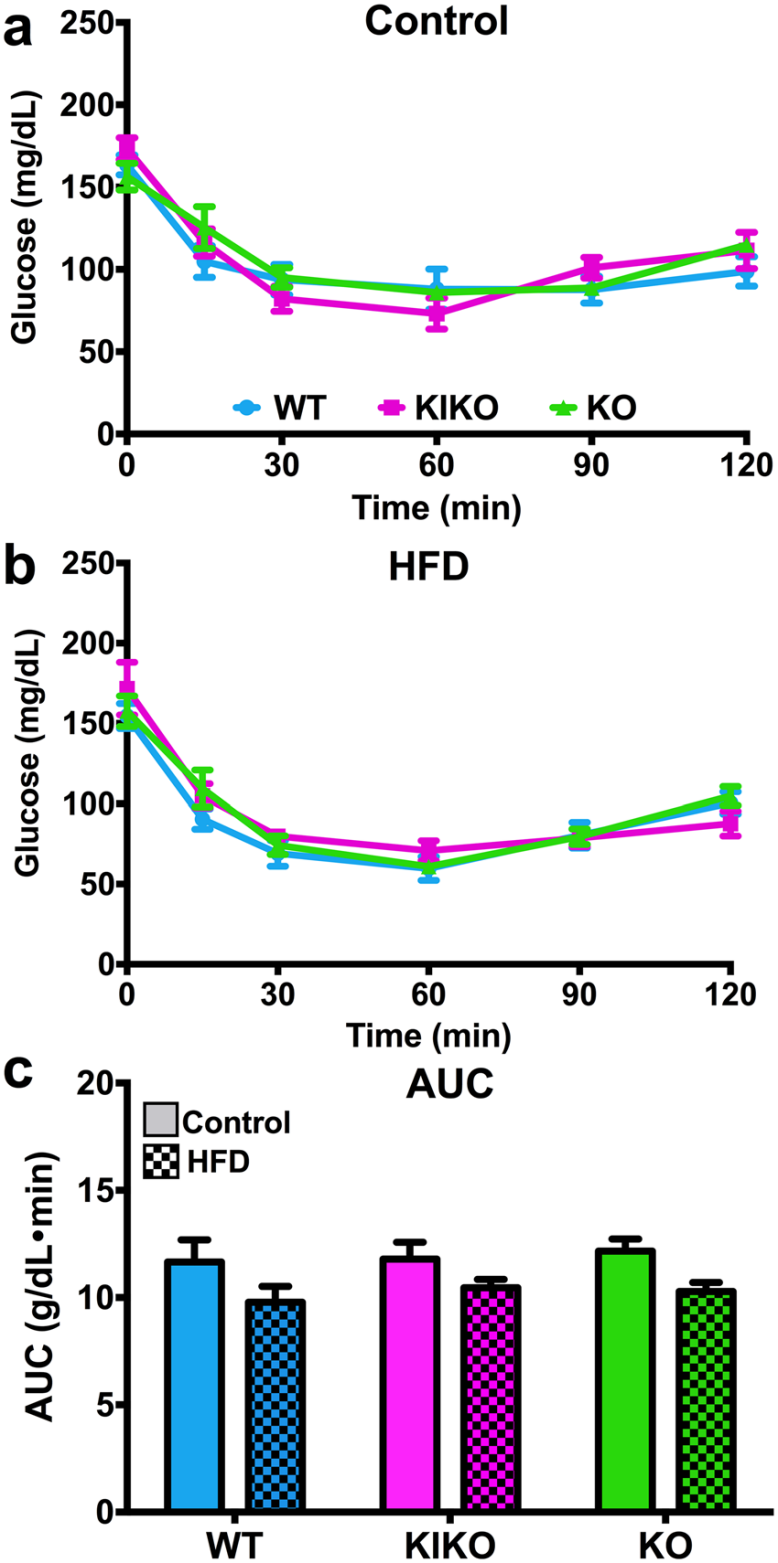
Insulin tolerance test (ITT) in adult females from all genotypes after 20 weeks of adult chow diet. Results from the ITT from (**a**) all genotypes from Control-fed dams and (**b**) all genotypes from HFD-fed dams. (**c**) AUC analysis for all genotypes from both maternal diets. (**a** and **b**) Data were analyzed by repeated-measures, multi-factorial ANOVA with *post-hoc* Newman-Keuls test. (**c**) Data were analyzed by a two-way ANOVA with *post-hoc* Newman-Keuls test. See Fig. 1 for information on treatment categories, sample sizes, and statistical comparisons (a/*/# = P < 0.05; b/**/## = P < 0.01; c/***/### = P < 0.001; d/****/#### = P < 0.0001).

To determine the effects of maternal HFD on glucose homeostasis, we conducted glucose and insulin tolerance tests on all females. For the GTT, all mice were fasted overnight (1700h – 0900 h). Fasting glucose levels, an indicator of a diabetic-like state, were not affected by genotype (Fig. 3a). There was no effect of maternal HFD on terminal blood triglycerides (non-fasted) (data not shown). Glucose tolerance was determined over 180 min following an ip injection of glucose (2 g/kg). Glucose clearance was slower in KO from Con-fed dams compared to WT and KIKO females at 60, 90, 120, and 180 min (Fig. 3b) and in KO from HFD-fed dams compared to WT and KIKO females at 30, 60, 90, and 120 min (Fig. 3c). Maternal HFD did not alter glucose clearance in any genotype (Fig. 3d), although maternal HFD augmenteded glucose clearance in WT at 30 min (P < 0.05, comparison not shown). Integral analysis of the area under the curve (AUC) illustrates the influence of genotype on glucose clearance (Fig. 3d). KO exhibited slower glucose clearance compared to WT, regardless of diet treatment (P < 0.05, P < 0.01, respectively). Insulin tolerance was measured over 120 min after an ip injection of insulin. In all genotypes, insulin-induced glucose clearance was not altered by maternal HFD (Fig. 4a–c; A comparison of all groups for the GTT and ITT is presented in Supplemental Figure S3). Therefore, the primary driver behind the inhibition of glucose clearance is the loss of ERE-independent actions by ERα, which has recently been elucidated in an adult HFD study with the same transgenic strains^37^.

### Hormones and inflammatory cytokines

**Figure 5:**
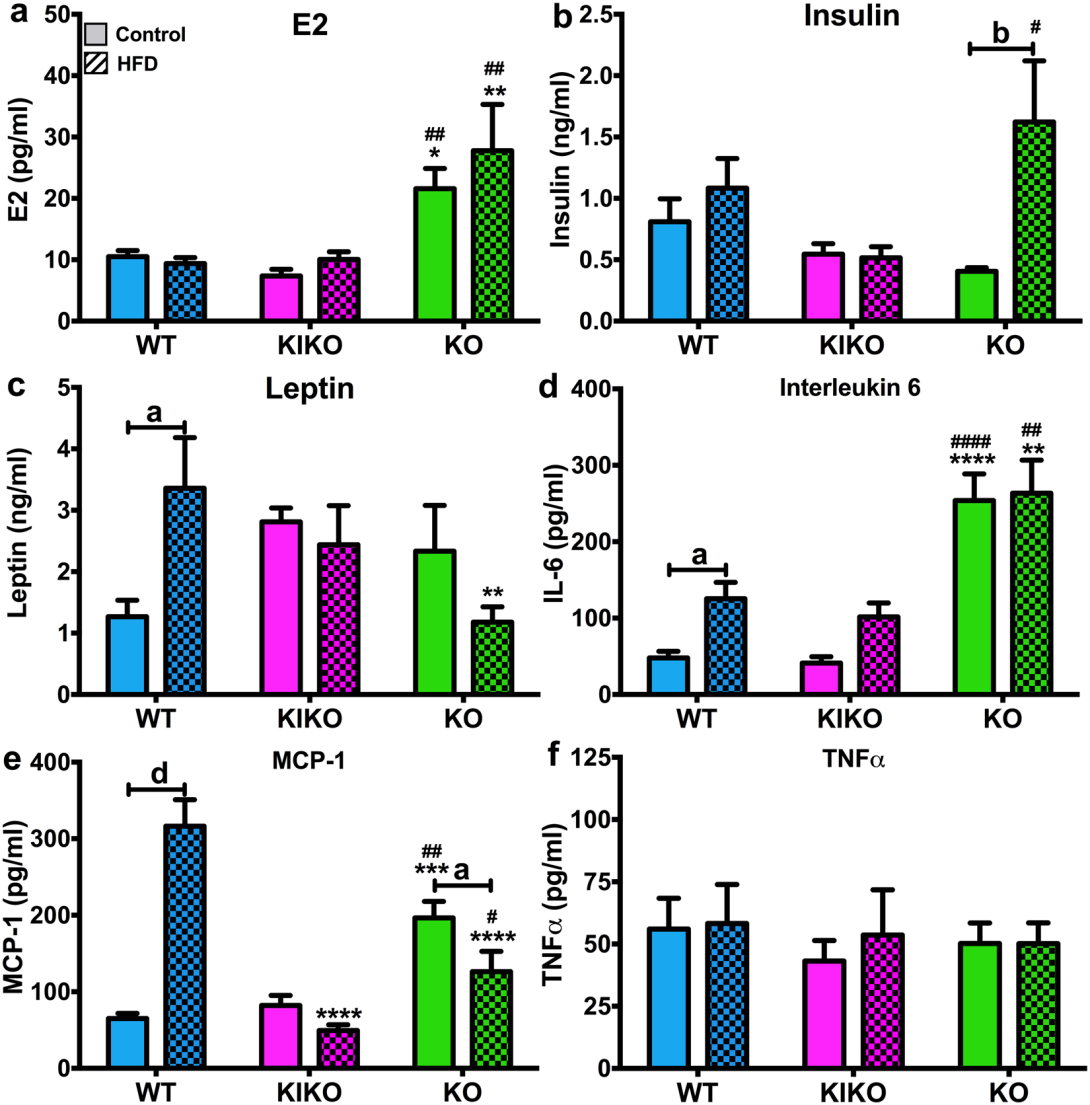
Peripheral peptide hormones and inflammatory cytokines from all genotypes after 20 weeks of adult chow diet. (**a**) Plasma levels of 17β-estradiol (pg/ml). (**b**) Plasma levels of insulin (ng/ml). (**c**) Plasma levels of leptin (ng/ml). (**d**) Plasma levels of IL-6 (pg/ml). (**e**) Plasma levels of MCP-1 (pg/ml). (**f**) Plasma levels of TNFα (pg/ml). Data were analyzed by a two-way ANOVA with *post-hoc* Newman-Keuls test. See Fig. 1 for information on treatment categories, sample sizes, and statistical comparisons (a/*/# = P < 0.05; b/**/## = P < 0.01; c/***/### = P < 0.001; d/****/#### = P < 0.0001).

To determine if maternal HFD alters endogenous E2 production, we measured E2 in all groups. E2 levels were not affected by maternal HFD and were higher in KO compared to WT and KIKO in both groups (Fig. 5a). To determine the effects of maternal HFD on leptin, insulin, and inflammatory cytokines, we analyzed plasma samples using multiplex assays. Maternal HFD did not alter plasma insulin levels in WT or KIKO. In contrast, maternal HFD produced hyperinsulinemia in KO, which expressed four times the plasma insulin as KO from Con-fed dams (P < 0.01; Fig. 5b), suggesting that ERE-independent signaling is protective against the effects of maternal HFD on insulin production. Maternal HFD increased plasma leptin in WT (P < 0.05), and plasma leptin in KO from HFD-dams were lower than WT (P < 0.01; Fig. 5c).

The selected inflammatory cytokines IL-6, MCP-1, and TNFα are all implicated in obesity^38^. Plasma IL-6 levels were primarily affected by genotype (Fig. 5d). Plasma IL-6 levels in KO were higher than WT and KIKO in control (P < 0.0001) and HFD (P < 0.01) groups, and IL-6 was elevated by maternal HFD in WT (P < 0.05). Plasma MCP-1 expression was affected by genotype and maternal HFD (Fig. 5e). KO from Con-fed dams expressed more MCP-1 compared to WT (P < 0.001) and KIKO (P < 0.01) and KO from HFD-fed dams expressed less MCP-1 compared to WT (P < 0.0001) and more than KIKO (P < 0.05). MCP-1 was also lower in KIKO from HFD-fed dams compared to WT (P < 0.0001). However, maternal HFD increased the levels of plasma MCP-1 in WT (P < 0.0001) and decreased plasma MCP-1 in KO (P < 0.05). Plasma TNFα levels were not affected by either genotype or maternal HFD (Fig. 5 f). Elevated levels of IL-6 and MCP-1 in KO females and in WT females from HFD-dams indicate chronic obesity and suggest that ERE-independent ERα signaling (KIKO) protects against systemic inflammation.

### Arcuate gene expression

**Figure 6:**
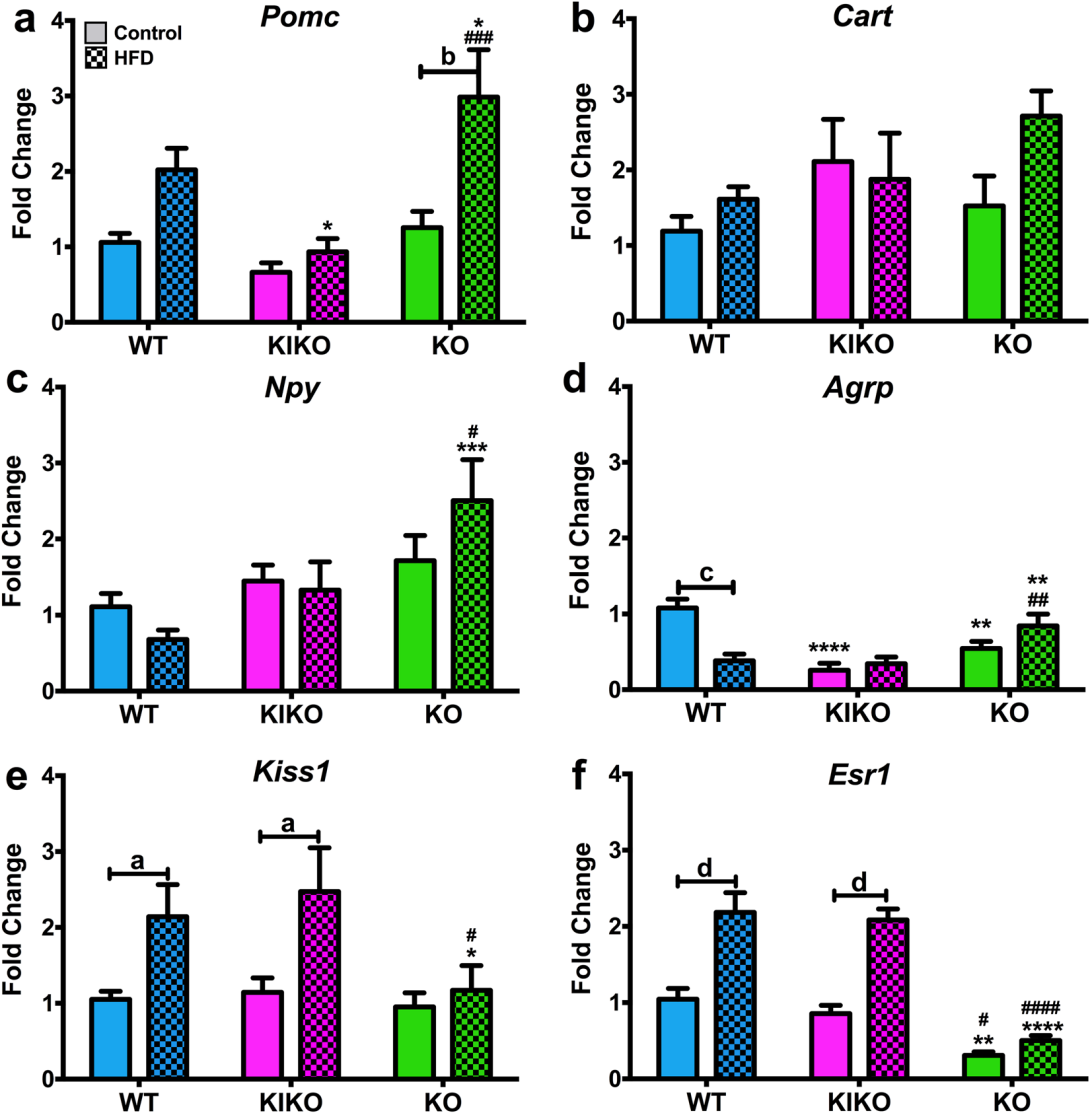
Arcuate gene expression in all genotypes after 20 weeks of adult chow diet. (**a**) *Pomc*; (**b**) *Cart*; (**c**) *Npy*; (**d**) *Agrp*; (**e**) *Kiss1*; and (**f**) *Esr1* (ERα) expression normalized to WT from Control-fed dams. Data were analyzed by a two-way ANOVA with *post-hoc* Newman-Keuls test within each genotype. See Fig. 1 for information on treatment categories, sample sizes, and statistical comparisons (a/*/# = P < 0.05; b/**/## = P < 0.01; c/***/### = P < 0.001; d/****/#### = P < 0.0001).

To determine if maternal HFD had a differential impact on ARC gene expression, we analyzed expression of selected genes involved in energy balance, including neuropeptides and hormone receptors^39^. For the anorectic neuropeptide, POMC, maternal HFD increased expression only in KO (Fig. 6a). *Pomc* expression was also dependent on genotype. Specifically, *Pomc* expression in WT from HFD-fed dams was higher than in KIKO (P < 0.05) and lower than in KO (P < 0.05). *Pomc* was also lower in KIKO from HFD-fed dams than KO (P < 0.001). *Cart* expression was not altered by genotype or maternal HFD (Fig. 6b). Expression of the orexigenic neuropeptide, NPY, was affected by genotype (Fig. 6c), with KO from HFD-dams expressing more *Npy* than both WT (P < 0.001) and KIKO (P < 0.05). *Agrp* expression was affected by genotype and maternal diet (Fig. 6d). Maternal HFD reduced *Agrp* expression in WT females. WT females from Con-fed dams also expressed more *Agrp* than both KIKO (P < 0.0001) and KO (P < 0.01). Conversely, KO females from HFD-fed dams expressed more *Agrp* than WT (P < 0.01) and KIKO (P < 0.01) females. Expression of *Kiss1*, a gene that has dual roles in reproduction and energy homeostasis^40, 41^, was augmented by maternal HFD in both WT (P < 0.05) and KIKO (P < 0.05) but not in KO (Fig. 6e). *Kiss1* expression in KO from HFD-fed dams was lower than both WT and KIKO (P < 0.05 for both). Expression of the ERα gene, *Esr1*, was dependent on genotype and maternal diet (Fig. 6 f). Receptor expression was reduced in KO compared to both WT (Con: P < 0.01; HFD: P < 0.0001) and KIKO (Con: P < 0.05; HFD: P < 0.0001) and was augmented by maternal HFD in WT females (P < 0.0001), as previously reported^42^, and in KIKO (P < 0.0001). Maternal HFD reduced arcuate expression of the insulin receptor (*Insr*) in WT (P < 0.05) (Supplemental Table S2) and KO from Con-fed dams expressed less *Insr* than WT (P < 0.001). Arcuate expression of the leptin receptor (*Lepr*) was augmented by maternal HFD in KIKO (P < 0.01) and was differentially expressed between the genotypes from HFD-fed dams (Supplemental Table S2).

### Liver gene expression

**Figure 7:**
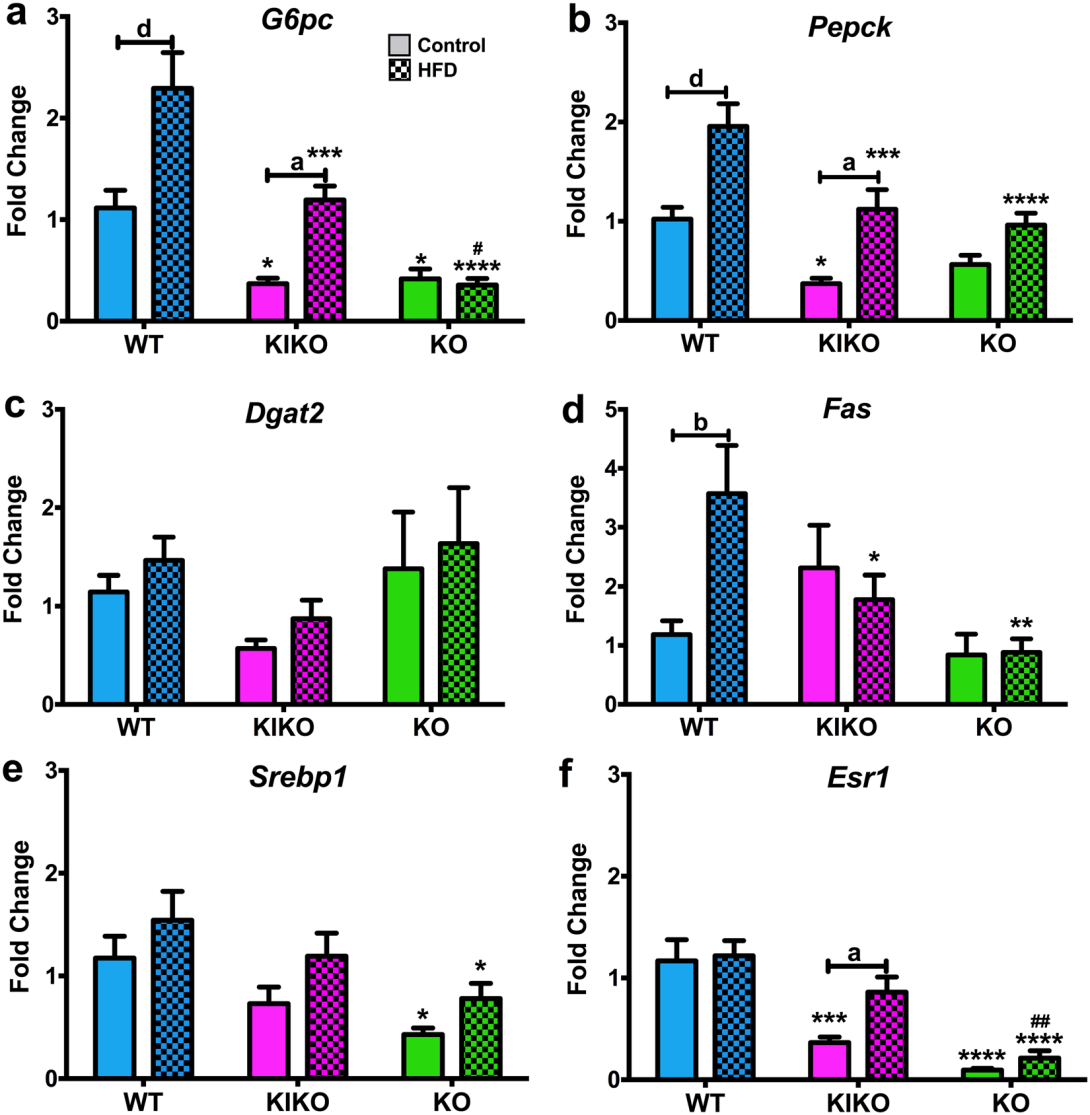
Liver gene expression in all genotypes after 20 weeks of adult chow diet. (**a**) *G6pc*; (**b**) *Pepck*; (**c**) *Dgat2*; (**d**) *Fas*; (**e**) *Srebp1*; and (**f**) *Esr1* (ERα) expression normalized to WT from Control-fed dams. Data were analyzed by a two-way ANOVA with *post-hoc* Newman-Keuls test within each genotype. See Fig. 1 for information on treatment categories, sample sizes, and statistical comparisons (a/*/# = P < 0.05; b/**/## = P < 0.01; c/***/### = P < 0.001; d/****/#### = P < 0.0001).

Because the effects of maternal HFD can also occur in peripheral organs that are involved in energy and glucose homeostasis^43–45^, we examined liver gene expression. Glucose-6-phosphatase (*G6pc*) expression, which controls hepatic glucose production^46^, was elevated by maternal HFD in WT (P < 0.0001) and KIKO (P < 0.05) females (Fig. 7a). Expression was dependent on genotype as both KIKO and KO expressed less *G6pc* than their WT counterparts. Phosphoenolpyruvate carboxykinase (*Pepck*), which is essential for gluconeogenesis, was differentially expressed between the genotypes and augmented by maternal HFD in WT (P < 0.0001) and KIKO (P < 0.05; Fig. 7b). KIKO and KO from HFD-fed dams expressed less *Pepck* than WT (P < 0.001 and P < 0.0001, respectively). Diacylglycerol O-acyltransferase 2 (*Dgat2*), which is an essential enzyme in the production of triglycerides^47^, was not affected by genotype or maternal HFD (Fig. 7c). Fatty acid synthase (*Fas*), which controls fatty acid production^48^, was augmented by maternal HFD in WT (P < 0.01), with lower expression in KIKO (P < 0.05) and KO (P < 0.01) from HFD-fed dams than WT (Figure 9d). Sterol regulatory element-binding protein 1 (*Srebp1*), a regulator of liver transcription for glucose, fatty acid, and lipid production^49^, was not altered by maternal HFD but was expressed less in KO than in WT (P < 0.05 for both; Fig. 7e). *Esr1* expression was elevated by maternal HFD in KIKO females (P < 0.05; Fig. 7f). *Esr1* was expressed at lower levels in KIKO (P < 0.001) and KO (P < 0.0001) from Con-fed dams compared to WT and at lower levels in KO from HFD-dams compared to WT (P < 0.0001) and KIKO (P < 0.01). Maternal HFD increased liver *Insr* expression in WT (P < 0.05) and KIKO (P < 0.01) and was expressed at lower levels in KIKO (P < 0.05) and KO (P < 0.01) from HFD-fed dams than WT. Maternal HFD reduced *Lepr* expression in WT (P < 0.01) and was expressed at lower levels in both KIKO (P < 0.0001) and KO (P < 0.0001) from Con-fed dams compared to WT (Supplemental Table S2).

## Discussion

Understanding the impact of maternal HFD on the development of central and peripheral mechanisms controlling energy homeostasis is key to addressing obesity and other metabolic diseases. Many studies in the field of maternal programming have examined male offspring mostly to avoid complications from the influence of circulating E2 on energy homeostasis in females during the estrous cycle, which is largely mediated by ERα. The role of ERα in the development of the reproductive functions of hypothalamus has previously been examined^50, 51^, yet its role in the development of energy homeostasis is largely unknown. Therefore, we set out to identify the importance of ERα in the development of female energy homeostasis by testing the hypothesis that females lacking ERα (KO) are more susceptible to the effects of maternal HFD. Instead, we found that KO from HFD-fed dams were not heavier than KO from Con-fed dams. This suggests that the disruption caused by the loss of ERα produces a “ceiling” effect and reduces the influence of maternal HFD. As previously reported, the ERE-independent ERα signaling present in KIKO females was sufficient to restore normal energy and glucose homeostasis compared to KO females. We found that it was also sufficient to restore the susceptibility to maternal HFD^29, 35^ because KIKO females, similar to the WT, were heavier after maternal HFD when fed a control diet. These data suggest that ERE-independent ERα signaling during development partially restores sensitivity to maternal HFD.

Recently, in an unpublished study, Flowers and colleagues^52^ (2014) presented evidence that KIKO and KO females are especially sensitive to diets low in phytoestrogens, which may confound the interpretation of our data. In our study, the dietary constituents both in the maternal and adult diets are not fully consistent, especially in regards to phytoestrogens. The control maternal diet used in the current study contains soy and an unknown concentration of phytoestrogens. In a 2007 study, phytoestrogens were measured at ~120 μg/g (ppt) chow in the same diet^53^, which is higher than the phytoestrogens in the HFD used in our study (Research Diets, personal communication). Furthermore, a previous study demonstrated that a lack of phytoestrogens in a diet fed to both dam and offspring produced heavier males and females at PND90, with more body fat and higher serum leptin levels, and a reduction in glucose clearance only in males^54^. In comparison, the adult diet used in our study was low in phytoestrogens (<75 ppm), but maternal HFD did increase body fat in WT and KIKO and plasma leptin levels in WT females.

In female rodents, E2 controls adipose deposition by decreasing visceral fat deposition primarily through an ERα-mediated mechanism^55^. In our study, the difference in fat mass between WT and KO was eliminated by maternal HFD as WT from HFD-fed dams were fatter than WT from Con-fed dams. These data suggest that the total loss of ERα reduces the developmental programming effects of maternal HFD on adipogenesis, which is restored by ERE-independent ERα signaling. However, ERα is not the only membrane-associated ER that has been implicated in the control of adiposity. GPER1 controls adiposity in females during DIO and may underlie some of the effects on adiposity found in the KIKO and KO females^56^.

Maternal HFD altered metabolism and activity by augmenting daytime V.O_2_, V.CO_2_, RER, and heat production (energy expenditure) in WT and heat production in KIKO and KO. These data suggest that the mechanisms of substrate utilization and energy expenditure are influenced during development, in part, by ERE-dependent and ERE-independent ERα signaling in females. The loss of ERα blocks the increase in carbohydrate utilization caused by maternal HFD during the day, which consequently blocks the nighttime increase in carbohydrate utilization. Thus, these effects on KO substrate utilization may play a role in the “ceiling effect” on obesity due to maternal HFD. Furthermore, the increase in energy expenditure after maternal HFD, which is found in the daytime in all genotypes, but only in WT and KIKO in the nighttime, may be a consequence of body weight gain in WT and KIKO^36, 57^. This suggests that the loss of ERE-independent signaling (in KO) during development results in an inhibition of the compensatory response in energy expenditure in heavier females.

Conversely, maternal HFD reduced activity in KO, widening the already prominent genotypic differences in activity. A recent study found that maternal HFD reduced exploratory behaviors and voluntary activity and increased anxiogenic behaviors in HFD-fed male and female mice^58^. We hypothesize that many of these effects are due to the mechanisms that ERα controls in the hypothalamus, both developmentally and during adulthood. In fact, selective deletion of ERα in neurons throughout the mouse brain produced an obese phenotype with an increase in food intake, a reduction in energy expenditure, increased adiposity, and suppressed activity^59^. In the same study, specific deletion of ERα in POMC neurons increased body weight, heat production, and activity^59^. Thus, the loss of ERα in select neurons during development produces phenotypes similar to those phenotypes produced by maternal HFD in KIKO and KO.

While activation of ERα in the liver is a primary pathway of E2 to control glucose production and insulin sensitivity, ERα also acts in adipose tissue and skeletal muscle^60, 61^. In our study, glucose clearance was reduced by the total loss of ERα signaling, as has been previously reported^29, 37^, maternal HFD did not have an impact on glucose clearance. Presumably, KIKO mice, like WT, retain the ability to shuttle glucose from the circulation due, in part, to the membrane-initiated ERα mechanisms that regulate glucose transporter type 4 (GLUT4) expression and insulin-induced trafficking to the membrane in skeletal muscle^60, 62, 63^. GLUT4 expression is increased through ERα activation in the extensor digitorum longus^63^, despite the lack of a consensus ERE in the GLUT4 promoter region, suggesting that ERE-independent signaling is key.

Similar to other maternal studies^64–66^, glucose homeostasis is not disrupted by maternal HFD in WT female offspring due to the protective effects of circulating estrogens activating both membrane-initiated and nuclear-initiated ERα signaling. However, the loss of total ERα signaling did not induce greater susceptibility as originally hypothesized. Likewise, maternal HFD did not alter insulin tolerance in any genotype, despite hyperinsulinemia in KO from HFD-fed dams, indicating that maternal HFD does induce insulin intolerance in the peripheral organs involved in glucose clearance. Interestingly, E2 replacement, both systemically and centrally (intracerebroventricular), in ovariectomized female rodents controls energy homeostasis, hepatic glucose production, and insulin sensitivity^67, 68^. Thus, we cannot ignore the potential role of ERα signaling in the hypothalamus when discussing the effects of maternal HFD on insulin and glucose homeostasis.

While maternal HFD did not have a clear effect on glucose homeostasis (fasting levels and glucose clearance), maternal HFD increased liver expression of *G6pc* and *Pepck* in WT and KIKO females. Elevated levels of these gluconeogenic enzymes suggest that hepatic glucose production is elevated in these genotypes from HFD-fed dams, which would require hyperinsulinemic-euglycemic clamp measurements. Interestingly, these genes were not upregulated in KO which may be evidence of protective hepatic glucose metabolism and contribute to the lower blood glucose levels. Furthermore, these genes were differentially expressed between the genotypes (WT expressed more than both KIKO and KO) and may produce a phenotype more susceptible to the effects of diet-induced obesity in adulthood.

Low-grade, elevated inflammation is a result of obesity due to increased production of inflammatory cytokines by adipose tissue. These cytokines are transported to organs that control metabolic processes e.g., liver, brain, and muscle^69–71^ and contribute to the developmental programming of maternal HFD^9, 72, 73^. In our study, maternal HFD augmented the peripheral inflammatory signals MCP-1 and IL-6 only in WT while MCP-1 and IL-6 was elevated in every KO group compared to WT and KIKO. Thus, the response to maternal HFD in WT includes an increase in cytokine production and may be a result of the increase in adiposity. However, this response in cytokine production to maternal HFD is lost in female mice that lack ERE-dependent ERα signaling despite increased adiposity. Furthermore, E2, through an ERα-mediated mechanism, enhances the HFD-induced increase in plasma IL-6 and TNFα levels in OVX female mice^74^. In our study, IL-6 was elevated in KO females, which were not insulin intolerant, from both Con-fed and HFD-fed dams. The elevation of IL-6, without other inflammatory signals, may promote glucose-stimulated insulin secretion from the pancreas^75–78^ and protect these females from further disruption to insulin homeostasis by maternal HFD.

ERα-mediated control of ARC gene expression is a primary mechanism to modulate hypothalamic and homeostatic functions^79^. Many studies have found that ARC neuropeptides are not altered by maternal HFD or obesity in adult male mice and rats^5, 7, 80^, while other studies have shown that maternal HFD stimulates and/or suppresses *Npy* and *Pomc* expression^81–83^. Due to the role that these ARC neuropeptides have in hypothalamic control of energy homeostasis, we hypothesized that maternal HFD would augment *Npy*/*Agrp* and suppress *Pomc*/*Cart*. However, we found elevated expression of the anorexigenic neuropeptide, *Pomc*, in KO females due to maternal HFD, which may result in a suppression in food intake. Conversely, expression of the orexigenic neuropeptide, *Agrp*, was reduced by maternal HFD in WT females, which may also result in a suppression in food intake. Interestingly, *Kiss1* expression, which has recently been implicated in the control of energy homeostasis in rodents^40, 84^, was elevated in WT and KIKO females by maternal HFD and may play a role in the effects of maternal HFD in these genotypes. Collectively, these data would indicate that the “ceiling effect” found in KO females may be due to an elevated anorexigenic gene expression profile and that both anorexigenic and orexigenic neuropeptides are impacted by maternal HFD, dependent on the availability of ERα signaling mechanisms.

Little is known about the interactions of maternal HFD and ERα on ARC gene expression, although hypothalamic ERα (and ERβ) protein expression is increased in female offspring from dams fed a HFD enriched with high levels of *n*-6 PUFA^42^. Our data are consistent with these findings, showing a two- to three-fold increase in *Esr1* expression in the ARC in WT and KIKO due to maternal HFD. The effect of these elevated levels of *Esr1* on energy homeostasis and on hypothalamic development is unknown but may be involved in ameliorating the effects of maternal HFD on neuroinflammation^72, 85^. Furthermore, because ERα mediates the actions of E2 on food intake and energy expenditure in the hypothalamus, the increase in ERα expression may be protective against the effects of maternal HFD in the WT and partially in the KIKO.

In conclusion, our study suggests that both ERE-dependent or ERE-independent ERα signaling during development influences the effects of maternal HFD on offspring energy and glucose homeostasis, inflammation, and gene expression. Presumably, the effects on energy expenditure and activity are central in origin, although further investigation is required. One potential mechanism is the epigenetic regulation of ERα in the brain by maternal HFD, which has previously been demonstrated with maternal behaviors and endocrine disruptors^86, 87^. Furthermore, ERα signaling regulates DNA methylation through the control of DNMT genes and other epigenetic factors in a variety of tissues^88–90^. The loss of ERα-induced epigenetic modifications along with the modulation of neurogenesis^31–33^ and neural stem cell proliferation and differentiation^34^ during development may abrogate the effects of maternal HFD. However, these data would indicate that at least some of these mechanisms involve ERE-independent ERα signaling since KIKO mice are susceptible to maternal HFD.

## Materials and Methods

### Animals

All animal treatments were in accordance with institutional guidelines based on National Institutes of Health standards and were performed with Institutional Animal Care and Use Committee approval at Rutgers University. Female wild-type (WT C57BL/6 J), ERα KO (KO), and ERα KIKO (KIKO) transgenic mice (provided by Dr. Ken Korach, NIEHS)^91, 92^ were selectively bred in-house and maintained under controlled temperature (23 °C) and photoperiod conditions (12/12 h light/dark cycle) with food and water *ad libitum*. WT/KO heterozygous males and females were mated to produce ERα KO females. Non-classical ERα knock-in heterozygous males (WT/KI) and WT/KO heterozygous females were crossed to generate KIKO females. WT females were generated from both colonies and used with their KIKO and KO littermates. At weaning, females were tagged and ear-clipped for genotyping. Genotype was determined by PCR of extracted DNA using previously published protocols^91, 92^.

### Maternal HFD Experimental Design

To determine the effects of maternal high-fat diet on energy homeostasis in female offspring, we modeled our experiment after a previous study that compared the effects of two maternal diets: a standard chow diet and a semi-purified high-fat diet^93^. Breeding WT/KO (*n* = 12/maternal diet) dams were fed either a standard breeder chow diet (Con, 25% fat kCal, 3.83 kcal/g, Lab Diet 5015; Lab Diet, St. Louis, MO, USA) or a high-fat diet (HFD, 45% fat kCal, 4.73 kcal/g, D12451; Research Diets, New Brunswick, NJ, USA) for 4 weeks prior to breeding with an untreated WT/KO or WT/KI male. Pregnant dams continued on the same diet for the duration of gestation and lactation (~10 weeks). HFD-fed dams gained more weight than the Con-fed dams prior to breeding (data not shown) but were not metabolically characterized during gestation or lactation to reduce the impact of stress on developmental programming^94^ and specifically on neuronal ERα expression^95^. After parturition, male pups were culled by postnatal day (PND) 4 to reduce the influence of litter size on offspring energy homeostasis. The average litter size was 9.1 ± 0.2 pups (n = 24) for Con-fed WT/KO dams and 8.8 ± 0.2 for HFD-fed WT/KO dams (n = 24). The average number of female pups per litter was 4.4 ± 0.2 for Con-fed WT/KO dams and 4.5 ± 0.2 for HFD-fed WT/KO dams. At PND 21, female pups from each litter were weaned and genotyped. Offspring were weaned onto a standard chow (13% kCal fat, 3.48 kcal/g, Lab Diet 5V75; low phytoestrogen, < 75 ppm) as the maternal control diet is specifically made to accommodate the high energetic needs of breeding females. At 5 weeks, all identified WT, KIKO, and KO females were weighed. Females were group-housed by genotype to reduce the social stress of single housing per IACUC protocols.

### Adult Offspring Experimental Design

From 5 to 25 weeks of age, females were weighed weekly. We did not monitor the estrous cycle as neither KO nor KIKO exhibit a normal estrous cycle, which makes it difficult to compare to WT^29, 30^. At the end of 25 weeks, body composition was measured in each female using an EchoMRI 3-in-1 Body Composition Analyzer (Echo Medical Systems, Houston, TX, USA) followed by a 48 h run in a Comprehensive Lab Animal Monitoring System (CLAMS) (Columbus Instruments, Columbus, OH, USA) to measure metabolic parameters and activity (X and Z plane). Females were then housed alone for one week to measure daily food intake. Afterward, a glucose tolerance test (GTT) was performed on each female. Females were fasted overnight (1700 h–0900 h) in a new cage. At the start of the test and 30 min after local anesthetizing of the tail with lidocaine, mice were placed in Plexiglass restrainers and tails were nicked to collect a baseline (time = 0) glucose reading using a glucometer (AlphaTRAK2). Immediately after baseline, females were injected intraperitoneally (ip) with a bolus of glucose (2.0 g/kg body weight) and individually housed in clean cages. Tail blood samples were collected at 15, 30, 60, 90, 120, and 180 min post-injection. After 180 min, all mice were returned to their home cages with *ad libitum* access to water and food. After sufficient recovery (~3 d), an insulin tolerance test (ITT) was performed after a 5 h fast in a similar manner as the GTT with an ip injection of insulin (0.75 units/kg). Blood samples were collected from the tail in individual cages at 15, 30, 60, 90, and 120 min post-injection. See Supplemental Figure S1 for a graphical illustration of the maternal and adult experimental design.

### Brain and Body Dissections

After sufficient recovery from the ITT (~1 week), females were decapitated after sedation with ketamine (100 µl of 100 mg/ml, ip) at 1000 h. Trunk blood was collected in a K^+^ EDTA collection tube and analyzed for triglyceride levels using a CardioChek (Polymer Technology Systems, Indianapolis, IN, USA). Plasma was prepared for peptide hormone and inflammatory cytokine analysis by adding a protease inhibitor, 4-(2-aminoethyl) benzenesulfonyl fluoride hydrochloride (AEBSF, 1 mg/mL, Sigma-Aldrich, St. Louis, MO, USA), to each collection tube. Samples were maintained on ice until centrifugation at 3,000 rpm for 10 min at 4 °C. Plasma was stored at −80 °C until analysis. Insulin, leptin, interleukin 6 (IL-6), monocyte chemoattractant protein 1 (MCP-1), and tumor necrosis factor α (TNFα) were determined by multiplex assay (MMHMAG-44K, EMD Millipore, Billerica, MA, USA).

Abdominal cavity was dissected for liver tissue (secondary lobe). Liver tissue was fixed in RNAlater (Life Technologies, Grand Island, NY, USA) and stored at –80 °C. Liver RNA was extracted using a standard TRIzol® extraction (Life Technologies) coupled with Macherey-Nagel NucleoSpin® RNA extraction and DNase-1 kit (Bethlehem, PA, USA). The brain was immediately extracted from the skull and rinsed in ice-cold Sorensen’s buffer for 30 sec. The brain was cut using a brain matrix (Ted Pella, Redding, CA, USA) into 1-mm thick coronal rostral and caudal blocks corresponding to Plates 42 to 47 and Plates 48 to 53, respectively, from *The Mouse Brain in Stereotaxic Coordinates* (Paxinos & Franklin 2008, 3rd Edition)^96^. Blocks of the basal hypothalamus (BH) were transferred to RNAlater (Life Technologies) and stored overnight at 4 °C. The rostral and caudal parts of the arcuate nucleus were dissected from slices using a dissecting microscope. Dissected tissue was stored at –80 °C. Total RNA was extracted from the combined rostral and caudal arcuate nucleus using Ambion RNAqueous-Micro Kits (Life Technologies) per the manufacturer’s protocol. Total RNA was treated with DNase I using the extraction kit protocol at 37 °C for 30 min to minimize any genomic DNA contamination. Liver and arcuate RNA quantity and quality were determined using a NanoDrop ND-2000 spectrophotometer (ThermoFisher, Waltham, MA, USA) and an Agilent 2100 Bioanalyzer and RNA Nano Chips (Agilent Technologies, Santa Clara, CA, USA). Only samples with RNA Integrity Number (RIN) > 7 were used.

Analysis of gene expression used standard protocols for quantitative real-time PCR (qPCR) as previously published^35^. Briefly, complementary DNA (cDNA) was synthesized using a standard Superscript III reverse transcriptase (Life Technologies) protocol: 5 min at 25 °C, 60 min at 50 °C, and 15 min at 70 °C. All primers were designed to span exon-exon junctions and synthesized by Life Technologies, using Clone Manager 5 software (Sci Ed Software, Cary, NC, USA). See Supplemental Table S1 for a listing of all the primer sequences used for quantitative real-time PCR (qPCR). Primers for *Esr1* were designed between exon 1 and 2, which is not deleted in the Ex3a ERα KO. qPCR amplification followed standard protocols for either PowerSYBR Green (Life Technologies) or Sso Advanced SYBR Green (BioRad, Hercules, CA, USA) master mixes on CFX-Connect Real-time PCR instrument (BioRad). All efficiencies were between 90–110%. The relative mRNA expression was calculated using the ΔΔC_T_ method utilizing a calibrator of diluted (1:20) cDNA from liver or BH of an untreated male. The geometric mean of the reference genes *Actb*, *Hprt*, and *Gapdh* was used to calculate δCq values. Quantification values were generated only from samples showing a single product at the expected melting point. All gene expression data were expressed as an *n*-fold difference relative to the calibrator^97^.

### Statistical Analysis

All data were expressed as mean ± SEM. Due to the occurrence of female WT, KIKO, and KO in each litter (~1 WT and 1 transgenic female/litter), each female represents one litter and all data were analyzed as such. All data were analyzed using Statistica 7.1 software (StatSoft, Tulsa, OK, USA) and by a two-way (maternal diet, genotype) or multi-factorial (maternal diet, genotype, time) ANOVA followed by a *post-hoc* Newman-Keuls test. GTT and ITT data were analyzed using repeated-measures, two-way ANOVA with a *post-hoc* Newman-Keuls test. All gene expression data were normalized to WT Control group for comparison across genotypes. All ANOVA statistics are presented in Supplemental Tables S3–S5. In all experiments, effects were considered significant at *α* ≤ 0.05.

### Data Availability

The datasets generated during and/or analyzed during the current study are available from the corresponding author on reasonable request.

## Electronic supplementary material

Supplementary Information
