# Supplementary material for: Loss of ERα partially reverses the effects of maternal high-fat diet on energy homeostasis in female mice
Source: Sci Rep. 2017 Jul 25;7:6381. doi: 10.1038/s41598-017-06560-x (PMC5526977; doi:10.1038/s41598-017-06560-x)
Supplement: Supplementary file 1 — Supplementary Information [file 41598_2017_6560_MOESM1_ESM.pdf]

Loss of ERα partially reverses the effects of maternal high-fat diet on energy homeostasis in female mice

Troy A. Roepke[1-4]*, Ali Yasrebi[1,2], Alejandra Villalobos[1], Elizabeth A. Krumm[1,2], Jennifer A. Yang[1,2]**, Kyle J. Mamounis[1,3]***

[1]Department of Animal Sciences, School of Environmental and Biological Sciences, Rutgers, The State University of New Jersey, New Brunswick, NJ. USA
[2]Graduate Program in Endocrinology and Animal Biosciences, Rutgers, The State University of New Jersey, New Brunswick, NJ. USA
[3]Nutritional Sciences Graduate Program, Rutgers, The State University of New Jersey, New Brunswick, NJ. USA
[4]New Jersey Institute for Food, Nutrition, and Health, Rutgers, The State University of New Jersey, New Brunswick, NJ. USA

Supplemental Data
Supplemental Figure S1

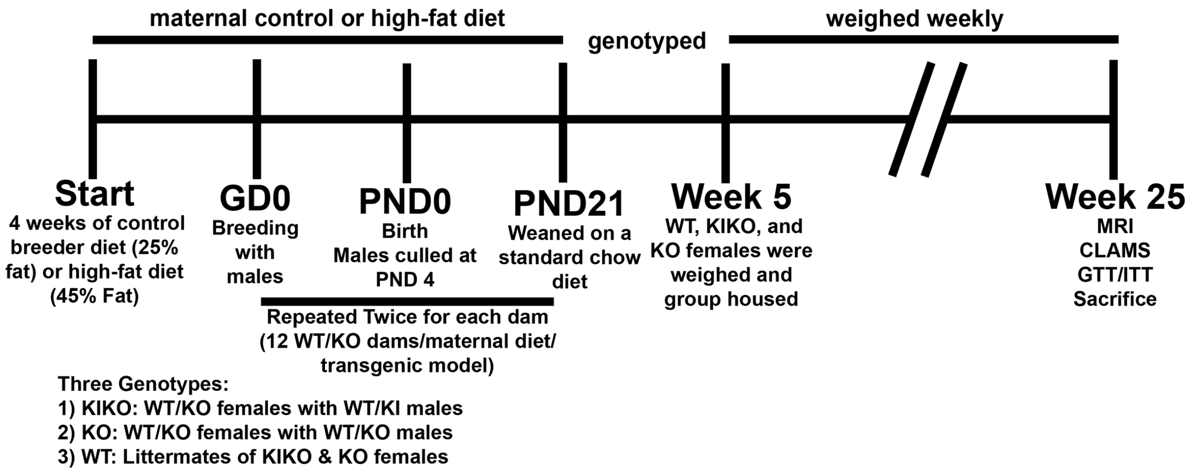

Supplemental Figure S1. Timeline of the experimental design for maternal high-fat diet and assessment of adult female offspring energy and glucose homeostasis.

Supplemental Table S1. Primer sequences

Gene Name	Forward Primer	Reverse Primer	Accession #
Actb	GCCCTGAGGCTCTTTTCCA	TAGTTTCATGGATGCCACAGGA	NM_007393.3
Agrp	CTCCACTGAAGGGCATCAGAA	ATCTAGCACCTCCGCCAAA	NM_007427.2
Cart	GCTCAAGAGTAAACGCATTCC	GTCCCTTCACAAGCACTTCAA	NM_013732
Dgat2	ACTCTGGAGGTTGGCACCAT	GGGTGTGGCTCAGGAGGAT	NM_026384.3
Esr1	GCGCAAGTGTTACGAAGTG	TTCGGCCTTCCAAGTCATC	NM_007956
Fas	GGGTTCTAGCCAGCAGAGTC	TCAGCCACTTGAGTGTCCTC	NM_007988.3
G6pase	GCCTCCTGTCGGATACAGAA	TGCACCGCAAGAGCATT	NM_008061.4
Gapdh	TGACGTGCCGCCTGGAGAAA	AGTGTAGCCCAAGATGCCCTTCAG	NM_008084.2
Hprt	GCTTGCTGGTGAAAAGGACCTCTCG AAG	CCCTGAAGTACTCATTATAGTCAAGGG CAT	NM_013556
Insr	GTGTTCGGAACCTGATGAC	GTGATACCAGAGCATAGGAG	NM_010568
Kiss1	TGATCTCAATGGCTTCTTGGCAGC	CTCTCTGCATACCGCGATTCCTTT	NM_178260
Lepr	AGAATGACGCAGGGCTGTAT	TCCTTGTGCCCAGGAACAAT	NM_146146.2
Npy	ACTGACCCTCGCTCTATCTC	TCTCAGGGCTGGATCTCTTG	NM_023456
Pepck	AGCGGATATGGTGGGAAC	GGTCTCCACTCCTTGTTC	NM_011044.2
Pomc	GGAAGATGCCGAGATTCTGC	TCCGTTGCCAGGAAACAC	NM_008895
Srepb1	TTGATAGAAGACCGGTAGCGC	CAGCTCAGAGCCGTGGTGA	NM_0114803

Supplemental Figure S2.

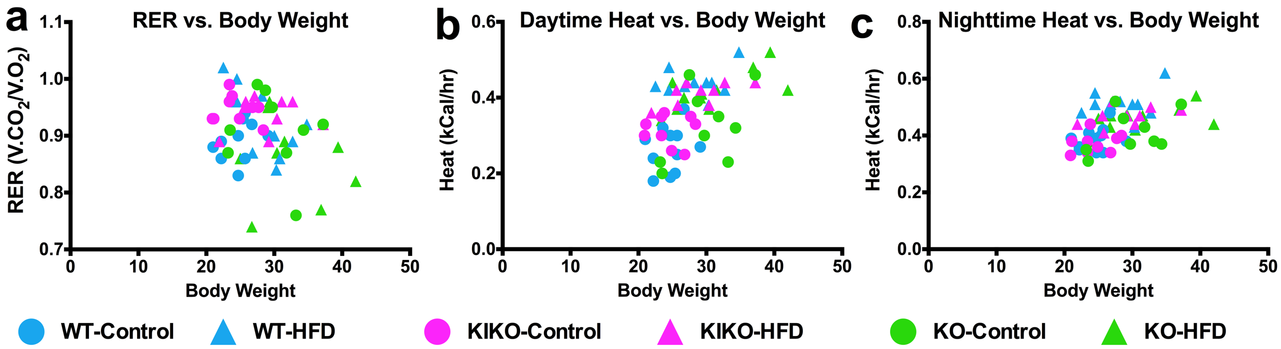

Supplemental Figure S2. A: Average daily RER as a function of body weight. ANCOVA: body weight: $F(1, 53) = 4.77$, $P = 0.033$; genotype: $F(2, 53) = 2.61$, $P = .083$; maternal diet: $F(1, 53) = .20$, $P = .656$; genotype*maternal diet: $F(2, 53) = 2.03$, $P = .141$. **B:** Daytime energy expenditure (kCal/hr) as a function of body weight. ANCOVA: body weight: $F(1, 53) = 9.71$, $P = 0.003$; genotype: $F(2, 53) = 0.20$, $P = .821$; maternal diet: $F(1, 53) = 53.42$, $P < .0000$; genotype*maternal diet: $F(2, 53) = 3.59$, $P = .034$. **C:** Nighttime energy expenditure (kCal/hr) as a function of body weight. ANCOVA: body weight: $F(1, 53) = 9.06$, $P = 0.004$; genotype: $F(2, 53) = 3.17$, $P = .050$; maternal diet: $F(1, 53) = 38.88$, $P < .0000$; genotype*maternal diet: $F(2, 53) = 2.92$, $P = .063$.

Supplemental Figure S3.

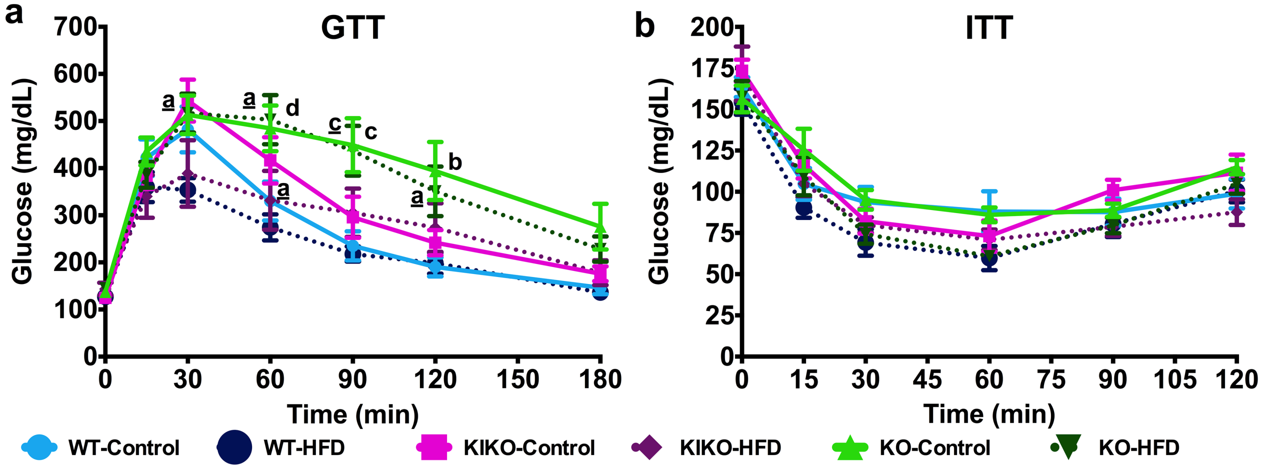

Supplemental Figure S3. A: GTT from all groups. **B:** ITT from all groups. Data were analyzed by a two-way ANOVA with *post-hoc* Newman-Keuls test. See Figure 1 for information on treatment categories, sample sizes, and statistical comparisons. Letters denote comparison between genotypes within the Con-fed dam group and underlined letters denote comparison between genotypes within the HFD-fed dam group (**a** = P < .05; **b** = P < .01; **c** = P < .001).

Supplemental Table S2. Arcuate and liver gene expression

Gene Name	Treatment	WT	KIKO	KO
ARC *Insr*	Control	1.21 ± 0.16	0.76 ± 0.13	0.37 ± 0.09 ***
	HFD	0.63 ± 0.09 **a**	0.74 ± 0.17	0.34 ± 0.09
ARC *Lepr*	Control	1.04 ± 0.10	1.25 ± 0.12	0.29 ± 0.05
	HFD	1.33 ± 0.27	2.75 ± 0.61 **b,****	0.46 ± 0.08 ***,####**
Liver *Insr*	Control	1.07 ± 0.12	0.51 ± 0.06	0.56 ± 0.08
	HFD	1.59 ± 0.21 **a**	1.10 ± 0.19 **b,***	0.90 ± 0.09 **
Liver *Lepr*	Control	1.10 ± 0.15	0.34 ± 0.06 ****	0.16 ± 0.03 ****
	HFD	0.66 ± 0.10 **b**	0.50 ± 0.16	0.26 ± 0.06 *

a = compared to Control within genotype, * compared to WT, # = compared to KIKO. (**a**/*/# = P < .05; **b**/**/## = P < .01; **c**/***/### = P < .001; **d**/****/#### = P < .0001

Supplemental Table S3. ANOVA statistics for each physiological endpoint

Endpoint	Genotype	Maternal Diet	Time	Interactions
weight 5 wks	F(2,54)=8.40, P<.001	F(1,54)=42.78, P<.0001		
weight 25 wks	F(2,54)=6.24, P<.01	F(1,54)=12.99, P<.001		
fat mass	F(2,55)=3.43, P<.05	F(1,55)=3.98, P<.05		
lean mass	F(2,55)=12.73, P<.0001	F(1,55)=5.33, P<.05		
V.O_2	F(2,108)=7.43, P<.001	F(1,108)=31.80, P<.0001	F(1,108)=60.66, P<.0001	
V.CO_2	F(2,108)=3.97, P<.05	F(1,108)=18.16, P<.0001	F(1,108)=59.62, P<.0001	g*m: F(2,108)=4.75, P<.05<br>m*t: F(1,108)=5.59, P<.05
RER	F(2,108)=9.75, P<.001		F(1,108)=36.04, P<.0001	g*m: F(2,108)=5.24, P<.01<br>m*t: F(1,108)=7.29, P<.01
Heat (energy expenditure)	F(2, 108)=3.13, P<.05	F(1,108)=70.34, P<.0001	F(1,108)=91.46, P<.0001	g*m: F(2,108)=4.63, P<.05<br>m*t: F(1,108)=7.77, P<.01
X	F(2,108)=23.76, P<.0001	F(1,108)=3.77, P=.0548	F(1,108)=183.9, P<.0001	g*t: F(2,108)=15.47, P<.0001
Z	F(2,108)=3.91, P<.05		F(1,108)=87.56, P<.0001	g*t: F(2,108)=4.00, P<.05
Triglycerides				
Fasting Glucose				
Control GTT	F(2,203)=15.42, P<.0001		F(6,203)=39.15, P<.0001	
HFD GTT	F(2,196)=23.76, P<.0001		F(6,196)=22.19, P<.0001	
GTT AUC	F(2,57)=10.49, P<.001			
Control ITT			F(5,162)=36.50, P<.0001	
HFD ITT			F(5,168)=57.23, P<.0001	
ITT AUC		F(2,55)=8.22, P<.01		
E2	F(2,36)=13.09, P<.0001			
insulin		F(1,51)=5.40, P<.05		F(2,51)=3.32, P<.05
leptin				F(2,51)=3.32, P<.05
IL-6	F(2,51)=30.62, P<.0001	F(1,51)=5.01, P<.05		
MCP-1	F(2,51)=16.94, P<.0001	F(1,51)=7.64, P<.01		F(2,151)=33.56, P<.0001
TNFα				

Interaction terms are defined as follows: g*m = genotype*maternal diet; m*t = time*maternal diet; g*t = genotype*time.

Supplemental Table S4. ANOVA statistics for arcuate nucleus gene expression

Arcuate	Genotype	Maternal Diet	Interactions
Agrp	F(2,53)=8.66, P<.001		F(2,53)=10.86, P<.001
Cart			
Esr1	F(2,53)=35.44, P<.0001	F(1,53)=45.40, P<.0001	F(2,53)=6.56, P<.01
Insr	F(2,53)=8.59, P<.001		
Kiss1		F(1,53)=8.89, P<.01	
Lepr	F(2,53)=15.02, P<.0001	F(1,53)=7.38, P<.01	
Npy	F(2,53)=6.66, P<.01		
Pomc	F(2,53)=7.14, P<.01	F(1,53)=12.16, P<.01	

Supplemental Table S5. ANOVA statistics for liver gene expression

Liver	Genotype	Maternal Diet	Interactions
Dgat2			
Esr1	F(2, 54)=32.04, P<.0001	F(1, 54)=4.09, P<.05	
Fas	F(2, 54)=4.83, P<.05		F(2, 54)=4.61, P<.05
G6pc	F(2, 54)=26.27, P<.0001	F(1, 54)=17.02, P<.001	F(2, 54)=5.70, P<.01
Insr	F(2, 54)=11.04, P<.0001	F(1, 54)=17.42, P<.001	
Lepr	F(2, 54)=21.19, P<.0001		F(2, 54)=4.78, P<.05
Pepck	F(2, 54)=16.23, P<.0001	F(1, 54)=30.92, P<.0001	
Srebp1	F(2, 54)=7.35, P<.01	F(1, 54)=5.70, P<.05	
